# Effect of gamma irradiation on properties of the synthesized PANI-Cu nanoparticles assimilated into PS polymer for electromagnetic interference shielding application

**DOI:** 10.1038/s41598-024-66356-8

**Published:** 2024-07-16

**Authors:** Mohamad Bekhit, E. S. Fathy, A. Sharaf

**Affiliations:** 1 Radiation Chemistry Department, National Center for Radiation Research and Technology, Egyptian Atomic Energy Authority, Cairo, Egypt; 2 Polymer Chemistry Department, National Center for Radiation Research and Technology (NCRRT), Egyptian Atomic Energy Authority (EAEA), Cairo, Egypt; 3 Radiation Engineering Department, National Center for Radiation Research and Technology (NCRRT), Egyptian Atomic Energy Authority (EAEA), Cairo, Egypt

**Keywords:** Nanocomposites, Polystyrene, Polyaniline, Copper nanoparticles, Gamma radiation, Electromagnetic interface (EM), Chemistry, Materials science, Nanoscience and technology

## Abstract

Conductive polymer nanocomposites for electromagnetic interference (EMI) shielding are important materials that can be combat the increasingly dangerous radiation pollution arising from electronic equipment and our surrounding environment. In this work, we have synthesized polyaniline-copper nanoparticles (PANI-Cu NPs) by the copper salt based oxidative polymerization method at room temperature and then added with different concentration (0, 1, 3 and 5 wt%) in polystyrene polymer forming PS/ PANI-Cu nanocomposites films by means of the traditional solution casting technique. The formed PANI-Cu NPs were investigated by UV/Vis spectroscopy, X-ray diffraction (XRD), transmission electron microscopy (TEM) and SEM/EDX elemental mapping techniques. On the other hand, the prepared PS/PANI-Cu nanocomposites films were evaluated by UV and SEM, the mechanical properties of the nanocomposites films were evaluated and showed an improvement by added PANI-Cu NPs up to 3 wt% and 50 kGy gamma exposure dose. The PS/PANI-Cu nanocomposites films were examined as electromagnetic interference shielding material. Electromagnetic shielding effectiveness of the produced nanocomposites were tested in the X-band of the radio frequency range namely from 8 to 12 GHz using the vector network analyzer (VNA) and a proper wave guide. All samples were studied before and after 50 kGy gamma-ray irradiation under the same condition of pressure and temperature. The results showed that the nanocomposites have improved shielding properties.

## Introduction

Recently, electromagnetic interferences (EMI) shielding materials are highly needed in electronic equipment and our surrounding environment to combat increasingly dangerous radiation contamination. Unfortunately, we all recently encounter EMI in our daily lives and it is expected to see a significant rise in the future owing to the increasing numbers of wireless devices such as mobile phones, GPS, Bluetooth, Wi-Fi and near field communication. The mitigation of the propagation of electromagnetic (EM) waves using shield materials (materials with electrical conductivity and/or magnetic properties) is very important and required. Nowdays, considerable effort has been made to develop electromagnetic interference (EMI) shielding materials. EMI shielding can be provided by blocking and preventing EM waves from passing through the electrical system either by absorption or by reflection of incident radiation. In the past, metals such as galvanized steel, aluminium, copper, nickel, tin-plated steel, zinc and silver were appropriately used for these purposes. Metals are a weight disadvantage, as today's electronic devices are faster, smaller and lighter. Recently, polymer-based EMI shielding materials are considered a promising due to the many advantages offered by the polymer such as lightness, low cost, corrosion resistance, flexibility and easy processability of polymers^1–3^. Recently, the nanomaterials and conducting polymers such as polyaniline (PANi), polypyrrole (PPy), polythiophene (PTh), and polydopamine are widely applied as electromagnetic wave absorbing materials^4–8^.

During the last decades, the polymeric materials have drawn considerable attention in various daily life applications, like in, electronics, sports, construction, packaging, transportation and medical uses, due to its excellent properties such as easy processing, flexibility and high mechanical strength. The polymeric composites materials can be achieved by adding two or more fillers (organic or inorganic), forming advanced materials with new properties that cannot be obtained through individual parts.

Recently, the combination of polymer and nanomaterials opens up new uses of the host polymers that called polymer nanocomposites. Nanocomposites are an evolving class of materials with extremely small phase dimensions (in the nanometer range). Due to their nanometer size advantages (large relative surface area to volume ratios), nanocomposites have distinctive properties over their conventional composite counterparts, thus providing new technology and commercial opportunities^9–11^.

Among the metal nanoparticles used, copper nanoparticles (CuNPs) and their oxide are one of the most common and widely used materials due to their outstanding properties such as excellent electrical and thermal conductivity, relatively high melting point and low electrochemical migration behavior. Moreover, they have low cytotoxicity and lower cost and this gives them high economic advantages^12–14^.

Polyaniline (PANI) is one of the most popular and important electrically conducting polymer due to the simplicity of preparation from cheap materials that make it superior to other conducting polymers. Also, PANI has excellent chemical stability to air oxidation, tunable electrochromic behavior and controllable electrical conductivity. Due to these advantages, PANI has found numerous applications in preparing light-weight batteries, heat transfer based nanofluids, conductive coatings, electrochromic devices, sensors, catalysis, textiles and electromagnetic shielding^15–23^. Unfortunately, PANI has poor capacity to form films and this drawback of PANI is solved in the field of blending or filling with other conventional polymers like polyvinyl alcohol (PVA)^24–27^, polypropylene (PP)^28,29^, polyethylene (PE)^30^ and polystyrene (PS)^31–33^. Polystyrene (PS) polymer is one of the most well-known thermoplastic polymers because of its properties such as low cost, easy processing good optical transparency, excellent thermal insulation, and long-term stability. Moreover, PS is a good hosting matrix for nanomaterials and has capacity to form films^34^. Using this blending method, the synergic amalgamation of enhanced electrical properties from PANI and improved mechanical properties from conventional polymers can be merged to produce a material with more potential applications in the electronics industry such as electromagnetic wave absorbing material^35–37^.

Modification of polymers by radiation is a distinct and promising technique for providing advanced polymeric materials, which can be characterized by specific physical and optical properties. This technique leads to specific changes in polymer properties due to induced crosslinking and/or chain-scission^38,39^. Exposure of polymeric material to ionizing radiation such as gamma rays, accelerated electrons, ion beams, and X-rays leads to the creation of highly reactive intermediates, free radicals, ions and excited species. These intermediates are transformed by different pathways such as disproportion, hydrogen absorption, rearrangements and/or formation of bonds. Generally, when a polymeric material is exposed to ionizing radiation, two mechanisms maybe happen: chain-scission and crosslinking. Crosslinking is a process in which the polymers chains are linked together and eventually the formation of a closed network system. Properties of polymer materials that can be enhanced by crosslinking include: mechanical properties, and performance at higher temperatures, often with an increase in the melting temperature. A competing process, called chain-scission. In this case, the polymer chains are broken and molecular mass decreases. During the irradiation of the polymers, both crosslinking and chain-scission occur simultaneously, but one is usually predominant, depending on the specific polymer, additives involved, and radiation dose. Moreover, radiation techniques can also modify other materials to enhance their physicochemical properties^40–42^.

In the present work, we have successfully fabricated PANI-Cu NPs by a simple, cheap and one pot route using copper salt based oxidative polymerization method at room temperature. Then, PANI-Cu NPs were dispersed in polystyrene with different concentration to prepare PS/PANI-Cu nanocomposites films using solution casting method. As a promise application for the PS/PANI-Cu nanocomposites films were its utilizing as electromagnetic shielding material to be used in different real life applications to decrease the increasing serious radiation pollution.

## Experimental

### Materials

Copper chloride dihydrate was obtained from Sigma-Aldrich. Aniline (C_6_H_5_NH_2_, M.wt = 93.13 g/mol) was obtained from TECHNO PHARM CHEM, INDIA. Polystyrene was obtained from Egyptian Styrene and Polystyrene Production Co. Egypt. Methanol was obtained from El Nasr Pharmaceutical Chemicals Co. Egypt. Chloroform was obtained from Sigma, USA.

### Preparation of PANI-Cu NPs

**Figure 1:**
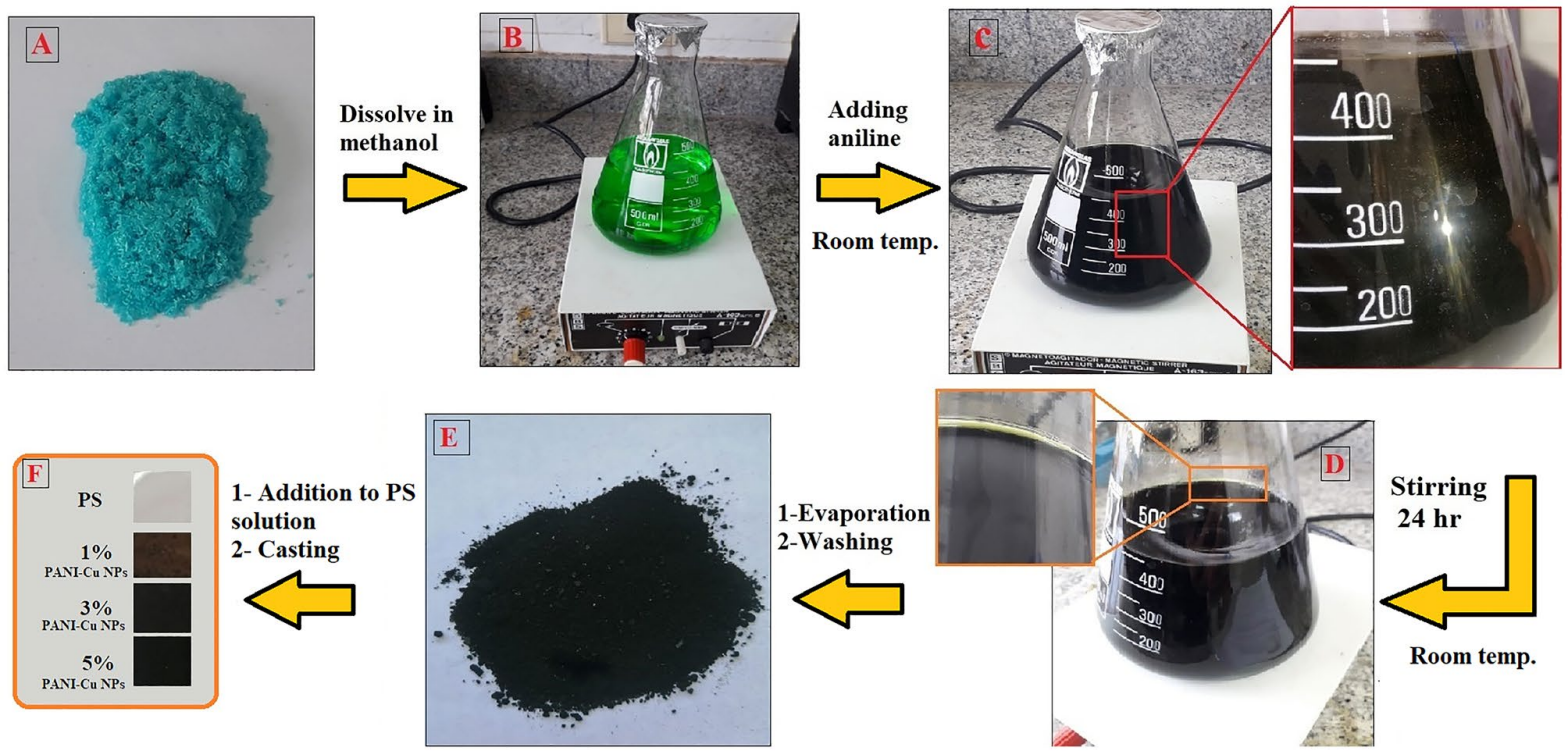
Procedures of PANI-Cu NPs preparation: (**A**) CuCl_2_ salt, (**B**) CuCl_2_-methanol solution, (**C**) initial formed PANI-Cu NPs, (**D**) Finally dispered PANI-Cu NPs, (**E**) PANI-Cu NPs powder and (**F**) PS/PANI-Cu Nanocomposites Films.

Oxidative polymerization in methanol was employed for the preparation of PANI-Cu NPs^43,44^. Firstly, copper chloride (0.2 M) was dissolved in methanol by means of a magnetic stirrer at room temperature for 30 min. Then, the pH value was adjusted to less than 4 using 1 M HCl. After that, aniline (0.4 M) was added into the copper chloride solution. The solution color changed immediately turned to dark red-brown colloid and kept under vigorous stirring for 24 h at room temperature. The solution color became brownish-green that confirming the successful preparation of Cu/PANI nanostructures (Fig. 1). The final solution was first evaporated to remove methanol. The precipitate was then washed with deionized water and 5 mM HCl to remove soluble oligomers and finally dried at room temperature.

### Fabrication of PS/PANI-Cu nanocomposites films

PANI-Cu NPs incorporated in a film of PS as host polymeric matrices was obtained by simple solution casting method. In a typical procedure, 1 g of polystyrene has been dissolved in 100 mL chloroform using a magnetic stirrer for 1 h. Afterthat, PANI-Cu NPs with different (0, 1, 3 and 5) wt% have been dispersed into polystyrene solution and kept under stirring for 24 h at room temperature to attain a uniform distribution of PANI-Cu NPs in polystyrene. Finally, nanocomposites films were obtained by casting the nanocomposites solutions in a glass Petri-dishes. The nanocomposites film’s thickness was controlled and adjusted during the casting process. After two days, the chloroform was completely volatilized and the nanocomposite films were stripped from the Petri-dishes. The fabricated nanocomposites films were exposed to 50 kGy gamma irradiation to study the effect of ionizing radiation on the prepared nanocomposites films. The radiation process performed in the National Centre for Radiation Research and Technology (NCRRT), Egyptian Atomic Energy Authority (EAEA), Egypt using 60Co radiation facility at room temperature and 0.8 kGy/h dose rate.

### Characterization of the nanocomposite

The X-ray diffraction analysis the synthesized PANI-Cu NPs was analyzed using an X-ray diffractometer (XRD, Shimadzu 6000) equipped with a Cu Kα (1.5418 Å) X-ray source. The infrared spectra was taken within the frequency range from 400 to 4000 cm^−1^ using Attenuated total reflectance-Fourier transform infrared (ATR-FTIR) spectroscopy (Vertex70, Bruker Optik GmbH, Ettlingen, Germany). Both size and shape of the synthesized nanoparticles was observed by Transmission electron microscopy (TEM) (a JEOL JSM-100 CX model instrument worked at 80 kV accelerating voltage). The PANI-Cu NPs sample were dispersed in ethanol and then dropped onto carbon film-coated copper grids. The surface morphology of pure PS and nanocomposites was observed by scanning electron microscope (SEM, ZEISS EVO-15, UK) operated at an acceleration voltage of 30 kV. For the SEM measurement, a thin layer of gold coated the fractured surfaces to avoid charging under the electron beam. The average molecular weight (M) of PANI was determined by the viscosimetric technique^45–47^. It was obtained by measuring the intrinsic viscosity[η] of polymer solution in DMF using an ubbelohde viscometer and applying the Mark–Houwink equation: [η] = K M^α^ where K and α are constants for DMF solvent at 25 °C (K = 2.5 × 10^−2^, α = 0.625). For measuring the mechanical properties, a dumbbell-shaped of PS/PANI-Cu nanocomposites films were tested using a tensile testing machine (Qchida computerized testing instrument, Dongguan Haida Equipment Co., Ltd. China) at a crosshead speed of 300 mm/min at 25 ± 2 °C. The ISO 527-2 was monitored. The average value of the mechanical measurements was taken via at least three samples. Direct current (DC) conductivity of the nanocomposites films were measured at room temperature. The sample was placed in a conductivity measuring cell in a sandwich configuration. HP 4280A C-V Plotter (USA) was utlized for measuring the conductivity of the samples under test.

### Electromagnetic interference assays

**Figure 2:**
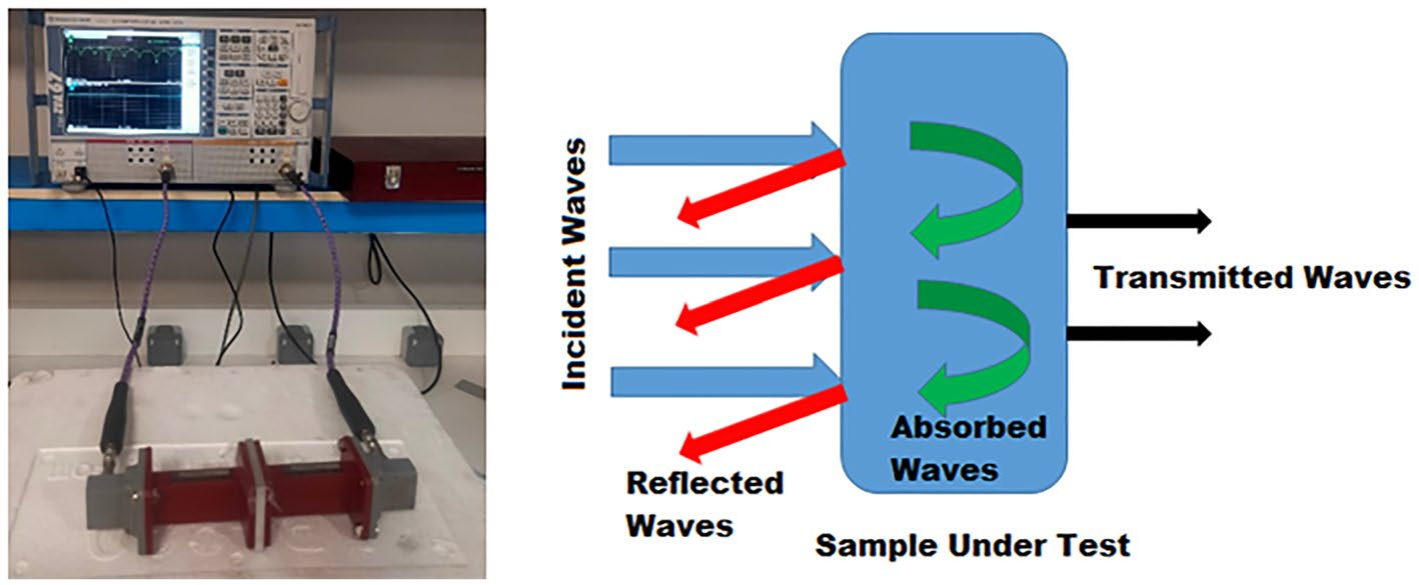
Electromagnetic Interface Shielding measuring concept and setup.

Shielding effectiveness for the unirradiated and 50 KGy irradiated nanocomposites was measured using a vector network analyzer and a proper wave guide. This measurement uses the R&S ZVA 67 VECTOR NETWORK ANALYZER operates in the range 10 MHz to 67 GHz and a wave guide operates in the X-band from 8 to 12 GHz. The VNA manufactured in Germany by Rohde and Schwarz GmbH & Co KG. The measuring setup was illustrated schematically in Fig. 2.

## Result and discussion

### Characterization of PANI-Cu NPs

**Figure 3:**
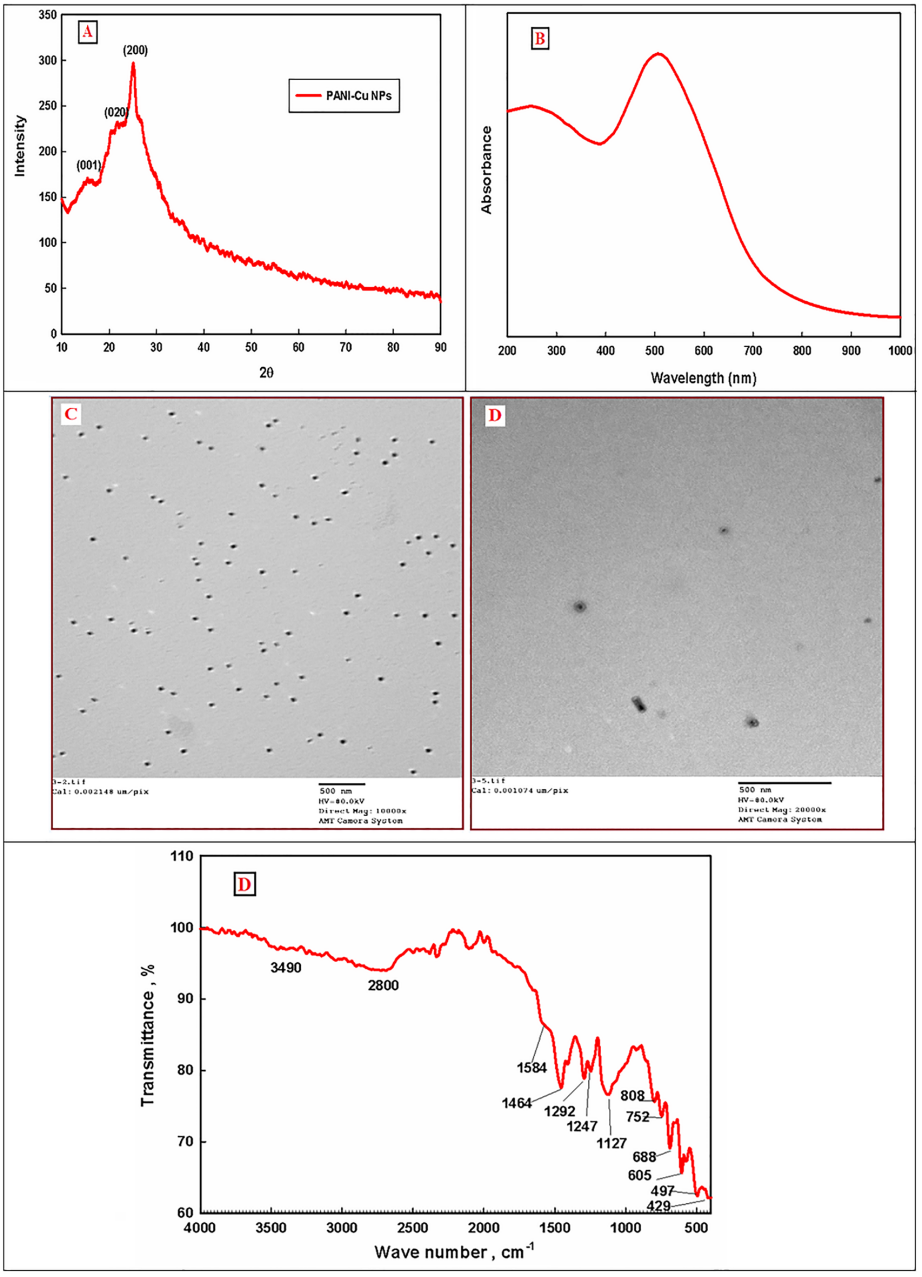
(**A**) XRD patterns, (**B**) UV–Vis spectrum, (**C**, **D**) TEM images with different magnification and FTIR Analysis of PANI-Cu NPs.

Figure 3A shows the XRD pattern of the as-prepared PANI-Cu NPs. The observed peaks 2θ angles 15.47°, 21.56° and 25.13° correspond to characteristic crystal planes (011), (020) and (200) of PANI respectively^48^. Also, the diffraction peaks of Cu NPs are not appeared and this can be attributed to the amorphous copper nanoparticles, coating effect of polyaniline and highly dispersed on the PANI support^49^. The more intense peak at 25.13° is associated with the enhanced face-to-face π–π stacking phenyl rings in PANI-Cu^50^. The UV–Vis absorption spectrum of the as-prepared PANI-Cu NPs was shown in Fig. 3B. As shown, the absorption spectra of PANI-Cu NPs have two absorption bands. The first band between 200 and 300 nm with wavelength maxima at 260 nm is due to π–π* transition of the benzenoid rings of the PANI backbone^51^, where the second broad band at 430–680 nm with wavelength maxima at 510 nm is due to the surface plasmone resonance of metallic copper nanoparticles^52,53^. Also, the broadening of the second band is attributed to the presence of n − π* transitions of imine nitrogen (–N=) of the quinoid ring in this region^51,54, 55^. The particle size and shape morphology of PANI-Cu NPs were performed by TEM and shown in Fig. 3C and D. It can be observed clearly that the PANI-Cu NPs have uniform spherical-shaped particles with mean diameter of about 25–35 nm. Magnified TEM image the PANI-Cu NPs indicates that these particles have a core shell structure consists of a black core of copper nanoparticles (8–13 nm) covered with a gray shell layer of PANI (15–23 nm)^56^. FTIR spectroscopy is a technique used for the functional groups identifications in the sample under test. FTIR spectrum of PANI-Cu NPs is shown in Fig. 3E. The peaks around 3490 and 2800 cm^−1^ are attributed to N–H and C–H stretching vibrations respectively. The peaks at 1584 and 1464 cm^−1^ corresponding to C=C stretching vibrations in quinoid ring (Q) and benzenoid ring (B), respectively^48,57^. Two peaks observed at 1292 and 1247 cm^−1^ assigned to the C–N stretching vibration mode in the quinoid and benzenoid imine units, respectively^58^. The peak at 1247 cm^−1^ is characteristic feature for conducting protonated form (C–N+• stretching vibration in the polaron structure)^56^. The broad and strongest absorption peak at 1127 cm^−1^ represents the in-plane C–H bending vibration of quinoid structure (vibrational mode structure is mode B–NH^+^=Q). This distinctive peak defined as electronic-like peak and measures the degree of delocalization of electrons on PANI and considered as characteristic peak of PANI conductivity^56,59, 60^. The peaks located at the lower wave number range (808–688 cm^−1^) are attributed to out of plane C–H bending vibration of the 1,4-disubstituted aromatic ring^43^. The data obtained from FTIR analysis established the oxidation of aniline monomer to PANI polymer and in good agreement with the previously reported data^61^. On the other hand, the three characteristic peaks observed at 429 cm^−1^, 497 cm^−1^, and 605 cm^−1^ can be ascribed to Cu–O stretching vibration^62^, suggesting partial oxidation of Cu NPs. The oxidation of CuNPs probably occurs because the extreme small size CuNPs are highly reactive due to the increased surface area^43^.

**Figure 4:**
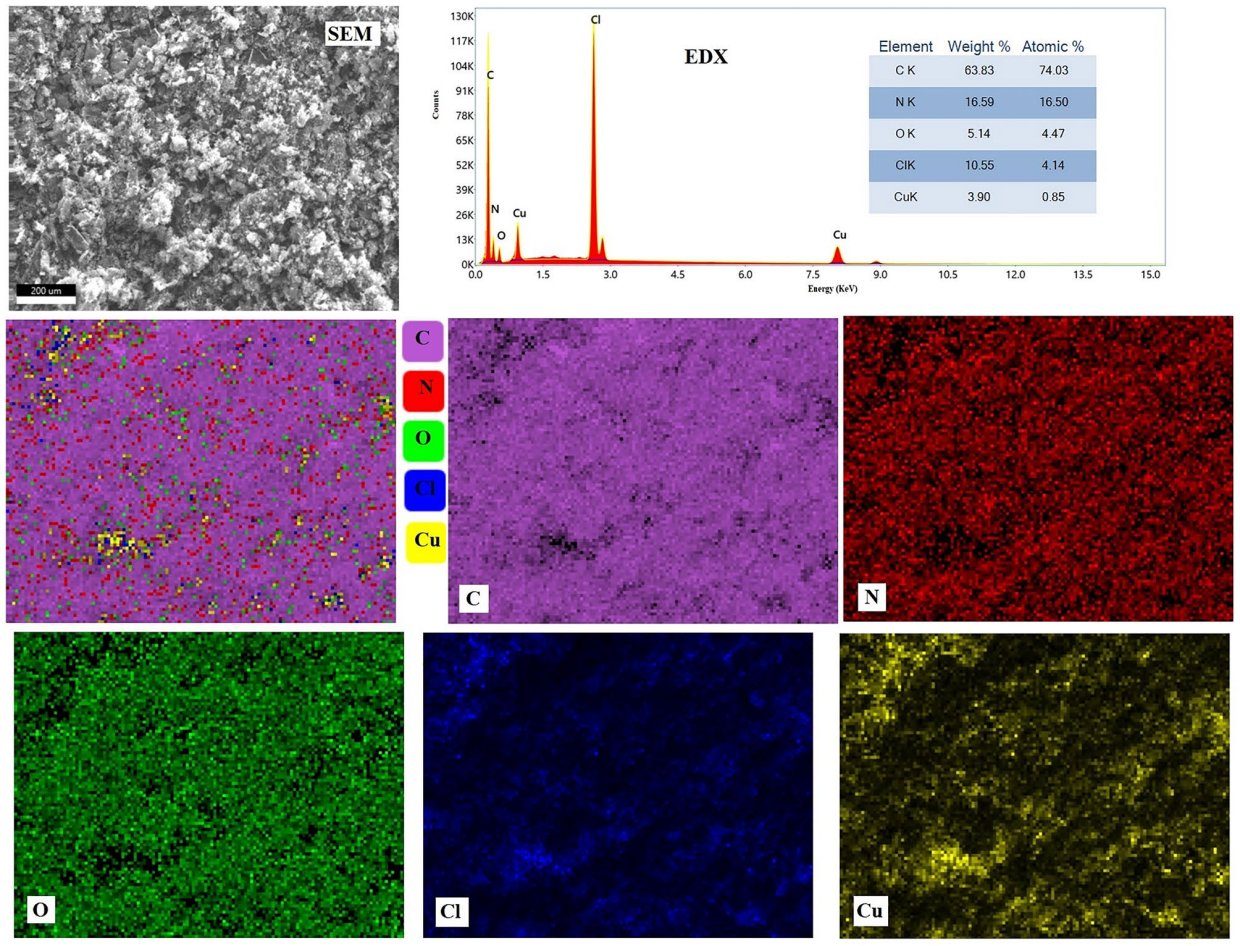
SEM/EDX elemental mapping images for PANI-Cu NPs.

EDX spectrum and mapping pattern of PANI-Cu are illustrated in Fig. 4. The EDX spectrum affirmed the presence of C, N, Cu, O and Cl elements in the composite and no foreign elemental peaks, verifying the purity of the PANI-Cu NPs. from the inset table the nanocomposite contained 3.9% of Cu (weight content). The elemental mapping images of PANI-Cu reveal that these elements are uniformly distributed on whole PANI-Cu NPs.

The mechanistic formation of PANI-Cu NPs occur through in situ oxidative polymerization technique where CuCl_2_ acts as an oxidizing agent and the amine nitrogen of aniline acts as sites for reducing the Cu^2+^ ions (reducing agent) and polymerization of aniline takes place simultaneously when aniline and CuCl_2_ solution are brought in contact with each other (reduction–oxidation polymerization process takes place) forming PANI-Cu NPs (see the following equation)^56,63, 64^. The fast formation of Cu nuclei during the early stage of the reduction leads to formation of Cu NPs has small sizes (< 10 nm).

The average molecular weight of the PANI/Cu nanocomposite was calculated on the basis of the Mark–Houwink equation to be 9388 Da. The calculated molecular weight in the current study was similar to which previously considered and reported in the literature^47^.
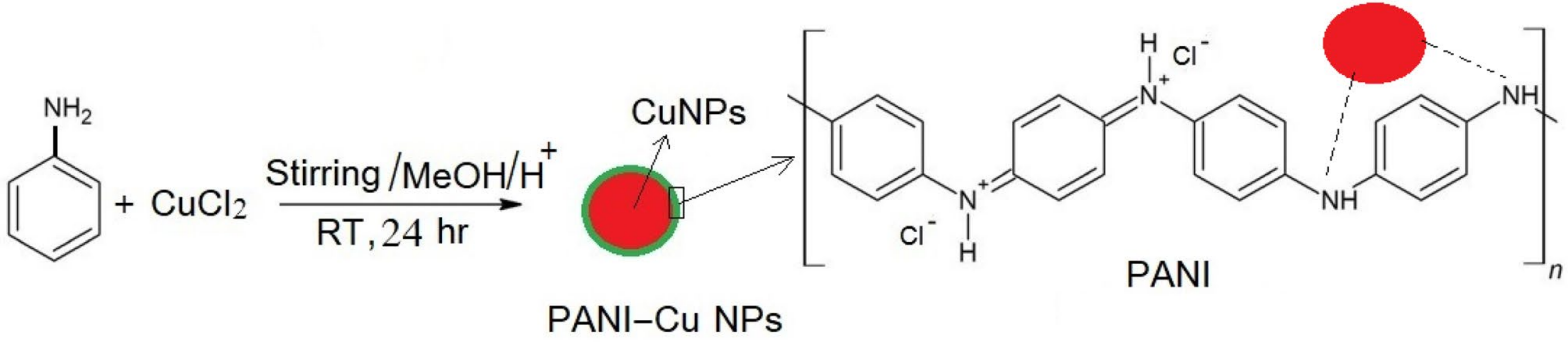


### Characterization of PS/PANI-Cu nanocomposite

#### UV–Vis analysis

**Figure 5:**
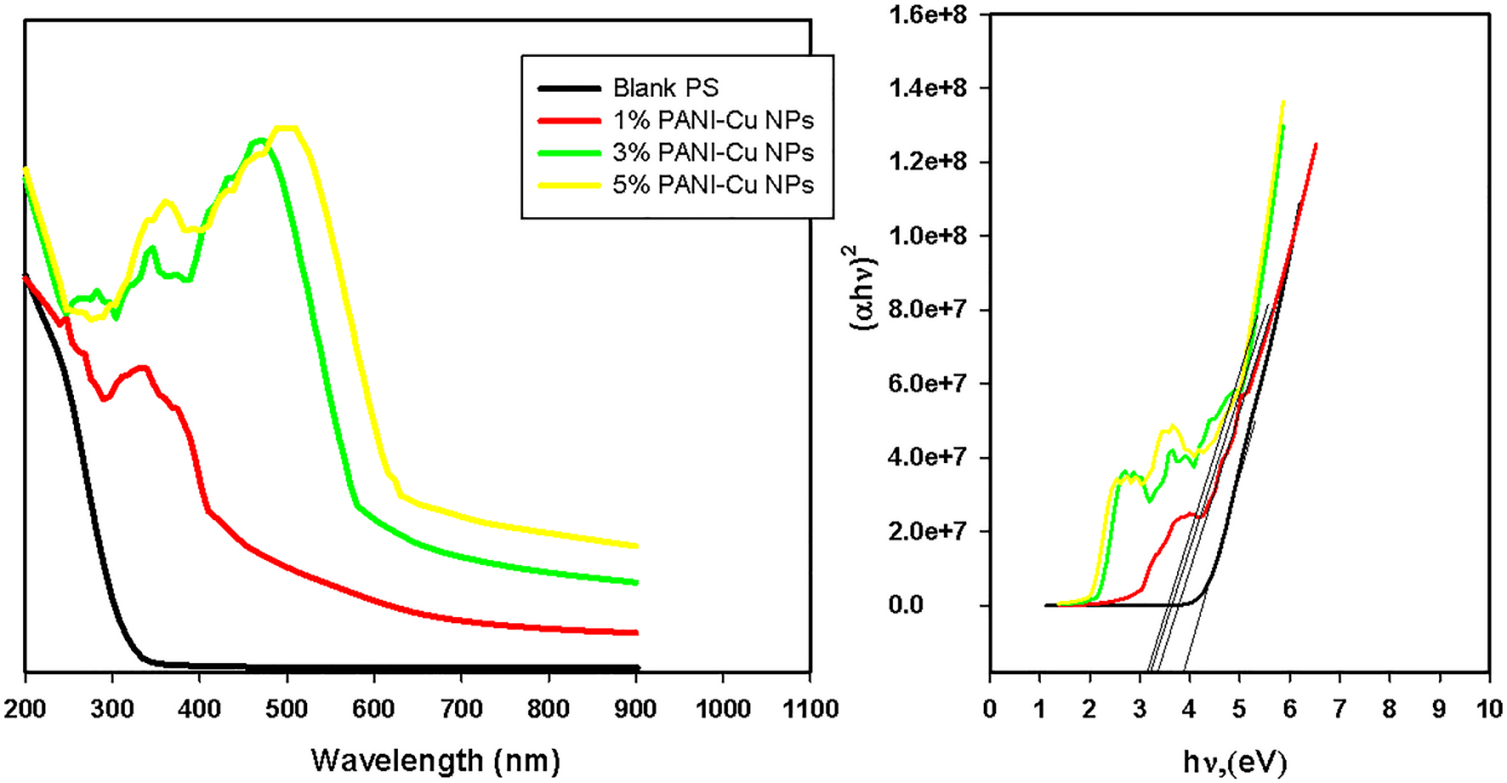
UV/Vis spectra of PS and PS/PANI-Cu Nanocomposites films.

**Figure 6:**
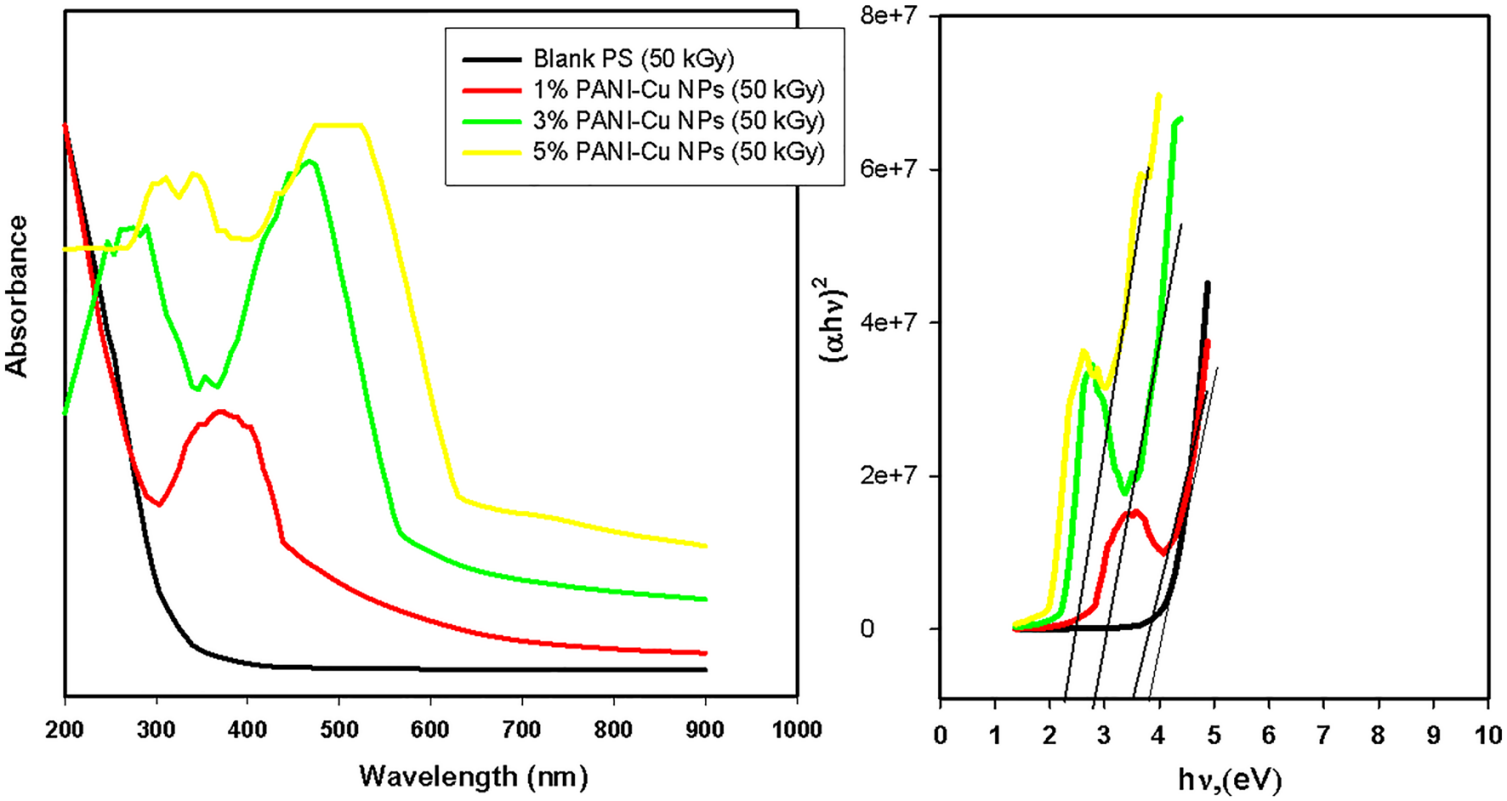
UV/Vis spectra of 50kGy irradiated PS and PS/PANI-Cu Nanocomposites films.

Figure 5 show the optical absorption spectra of PS and PS/PANI-Cu NPs nanocomposite films as a function of the PANI-Cu NPs. It was seen that in pure PS there is no absorption peak, and absorption peaks can be seen in PS/PANI-Cu nanocomposite films which indicative of the existence of PANI-Cu NPs. Also, it can be seen that there is a red shift in the absorption peaks with increase in intensity due to increasing the amount of PANI-Cu NPs which meaning the increasing the content of PANI. After gamma irradiation, an enhancement in the optical properties is observed (Fig. 6). The optical energy gaps (Eg) values of PS and PS/PANI-Cu nanocomposite films were estimated according to Tauc’s equations (αhʋ = B(hʋ − Eg)^n^) where *α* is the absorption coefficient, *h*ʋ is the energy of the incident photon and B is a constant. The direct optical energy gaps values were estimated by plotting the value of (*αh*ʋ)^2^ versus photon energy (*h*ʋ)^65^. The estimated values of the bandgap are presented in Table 1. From results, the values of the optical energy band gap of PS decreased with increasing concentration of PANI-Cu NPs and gamma ray irradiation. The decrease of optical band gap energy reflects the role of PANI-Cu NPs in modifying the electronic structure of the polymeric matrix^53,66^. On the other hand, the decrease of band gap with the subjection to gamma rays is attributed to increase the number of energy localized electronic states between the valence and conduction bands related to the subjection to gamma radiation where the chains becoming more and more cross-linked with one another as a result of subsequent irradiation^67–69^. In other words, when polymers exposed to the gamma radiation, there are many events occurs such as free radicals formation, polymer chain scission, unsaturation, cross-linking and/or carbon clusters formation due to hydrogen release from polymer chain. These effects increase the structural disorder that reduce the optical energy band gap and consequently increase in the conductivity of the irradiated samples^70^.

**Table 1:** Estimated values of optical energy gap obtained from Tauc equation.

Sample	E_g_ (eV) of unirradiated samples	E_g_ (eV) of 50 kGy-irradiated samples
**PS**	3.92	3.83
PS/1%PANI-Cu nanocomposites	3.54	3.52
PS/3%PANI-Cu nanocomposites	3.25	2.85
PS/5%PANI-Cu nanocomposites	3.16	2.26

#### Mechanical measurements

**Figure 7:**
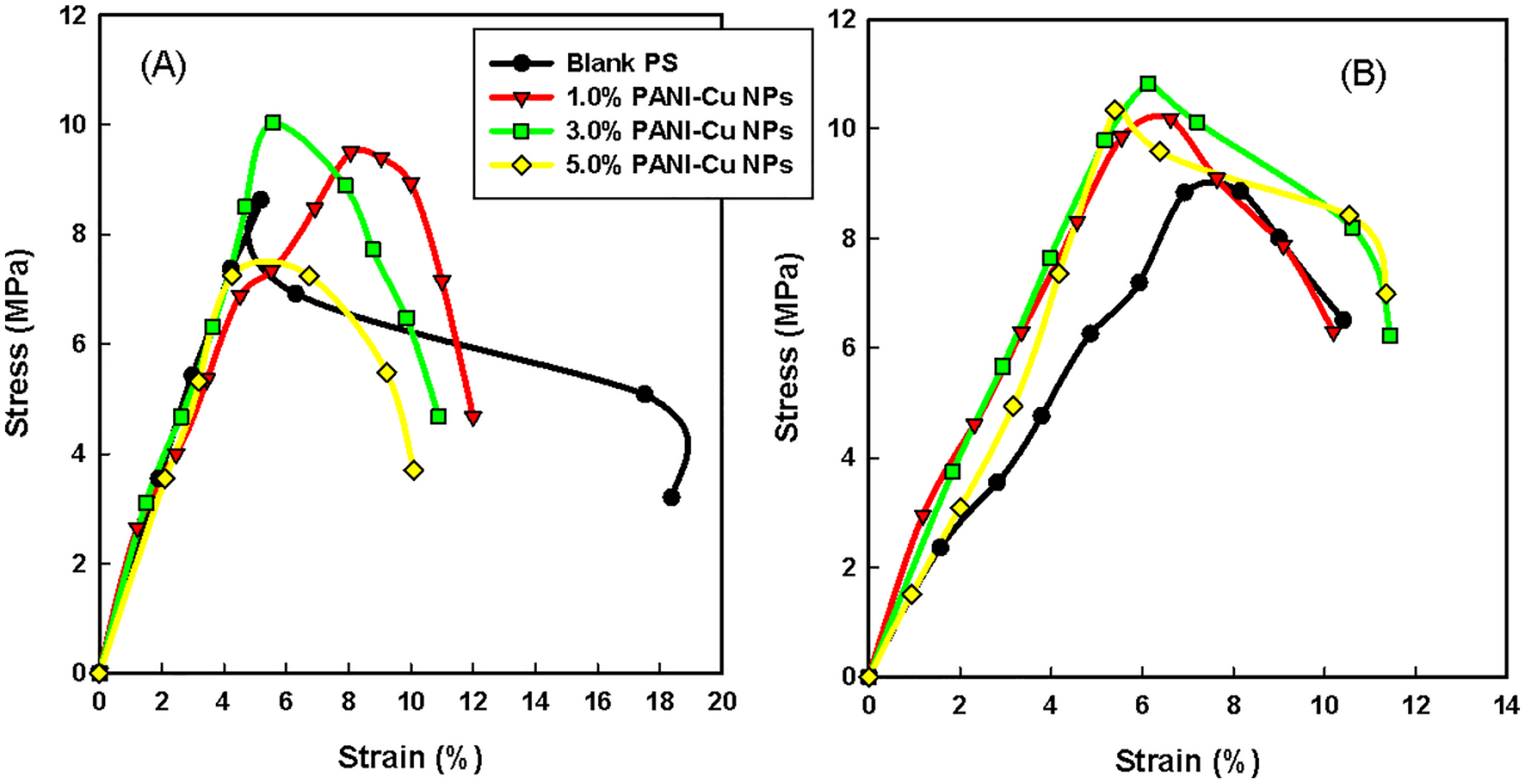
Stress–strain curves of (**A**) unirradiated and (**B**) 50 kGy irradiated PS and PS/PANI-Cu nanocomposites.

**Figure 8:**
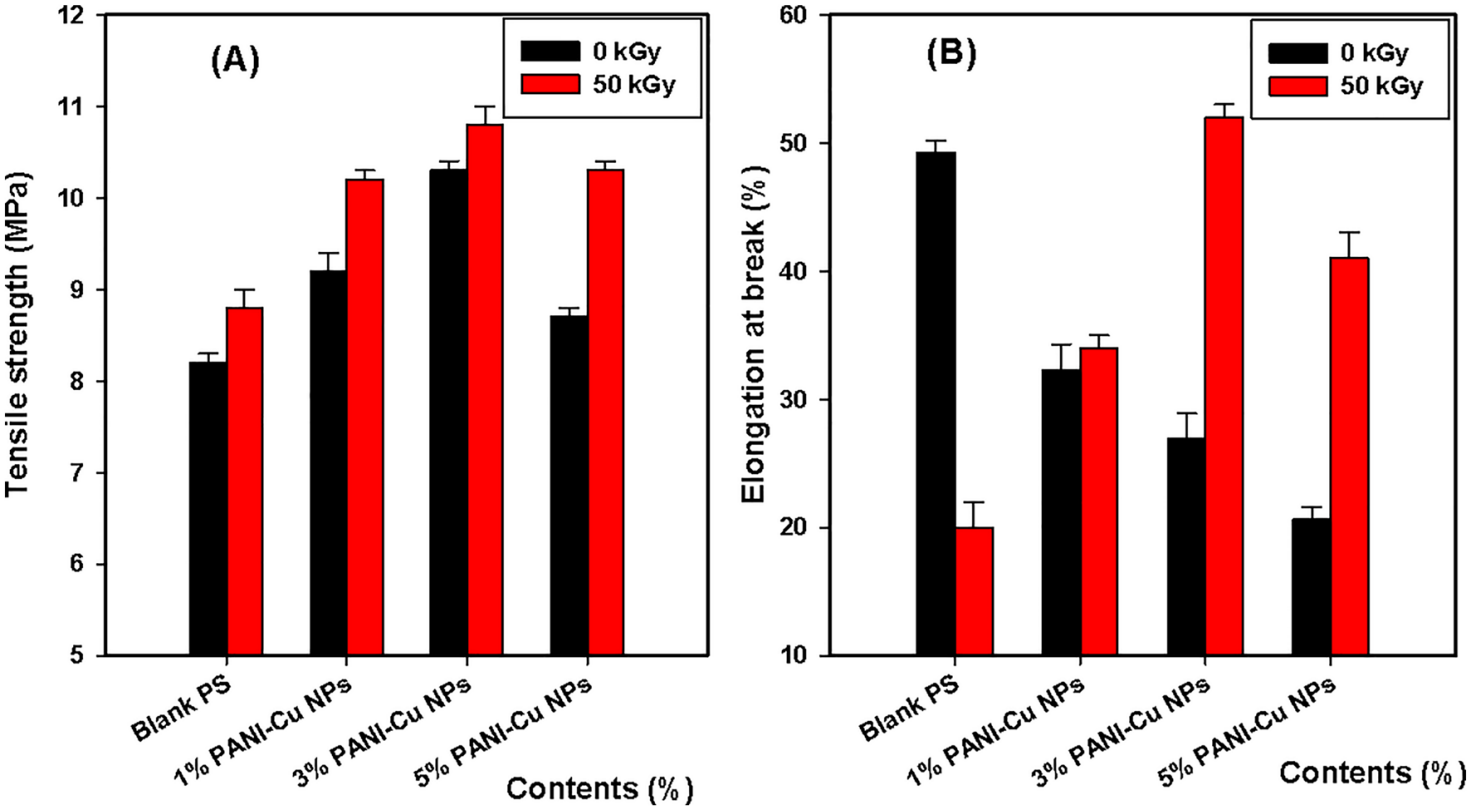
(**A**) Tensile strength (MPa), (**B**) Elongation at break (%) of PS and PS/PANI-Cu nanocomposites exposed to 50 kGy irradiation.

Figure 7A and B shows the stress–strain behavior of unirradiated and irradiated polystyrene (PS) and PS/PANI-Cu nanocomposites. From Fig. 7A, the tensile stress (TS) of PS increases with the PANI-Cu nanoparticle contents and 3.0% of the filler loading recorded the best. The detected behavior proposed that, by increasing PANI-Cu content, samples regularly lose the polymer chain mobility most feasibly because of the formation of specific polymer–nanoparticle interfaces. On the other hand, it is obvious that from Fig. 7B, the TS of the nanocomposites increases with applied irradiation dose, 50 kGy, indicating that crosslinking is the predominant^71^. Whereas, the elongation at break (%) represents anisotropy behavior. The data attained in the stress–strain curves were used to produce Fig. 8.

Figure 8A and B displays the results of tensile strength (TS) and elongation at break (E%) respectively, with an increase of polyaniline (PANI) nanoparticles content into Polystyrene (PS). The TS enhanced with an increase of PANI nanoparticle content up to 3.0 wt% due to the homogenous distribution and the better interaction between PS and PANI nanoparticles.After that, with the increase of polyaniline above 3.0 wt%, a decrease in the tensile strength values occurs. Where the tensile strength of PS decreased with the increase of polyaniline content at 5.0 wt% due to its fragility. For irradiated samples, up to 3.0 wt% loading of PANI, the TS of PS slightly increased with PANI nanoparticles and also reveals slightly superior to unirradiated ones. Whereas, the irradiated specimens of PS/PANI (5.0 wt%) revealed observed enhancement of TS over their counterpart unirradiated samples. Consequently, the gamma irradiation assisted the interfacial linkage between PS and PANI nanoparticles. The last consequence agrees with what was previously published^72^.

Elongation at break shows on the Fig. 8B of native PS was decreased dramatically when subjected to 50 kGy irradiation because of the rigidity and the loss of elasticity of the pristine PS caused by irradiation. Furthermore, the elongation at break of irradiated PS samples was improved with PANI nanoparticle intrusion, especially at 3.0 and 5.0 wt% of PANI filling. The tendency of extreme enhancement in elongation at break of PS/PANI with irradiation dose, at 50 kGy, is obvious. This effect could be related to the steady creation of radiation-induced crosslinking at this dose, which permits the pristine PS to undergo strain-induced crystallization^73^. The last result is consistent with what was previously published^74^.

#### Scanning electron microscope

**Figure 9:**
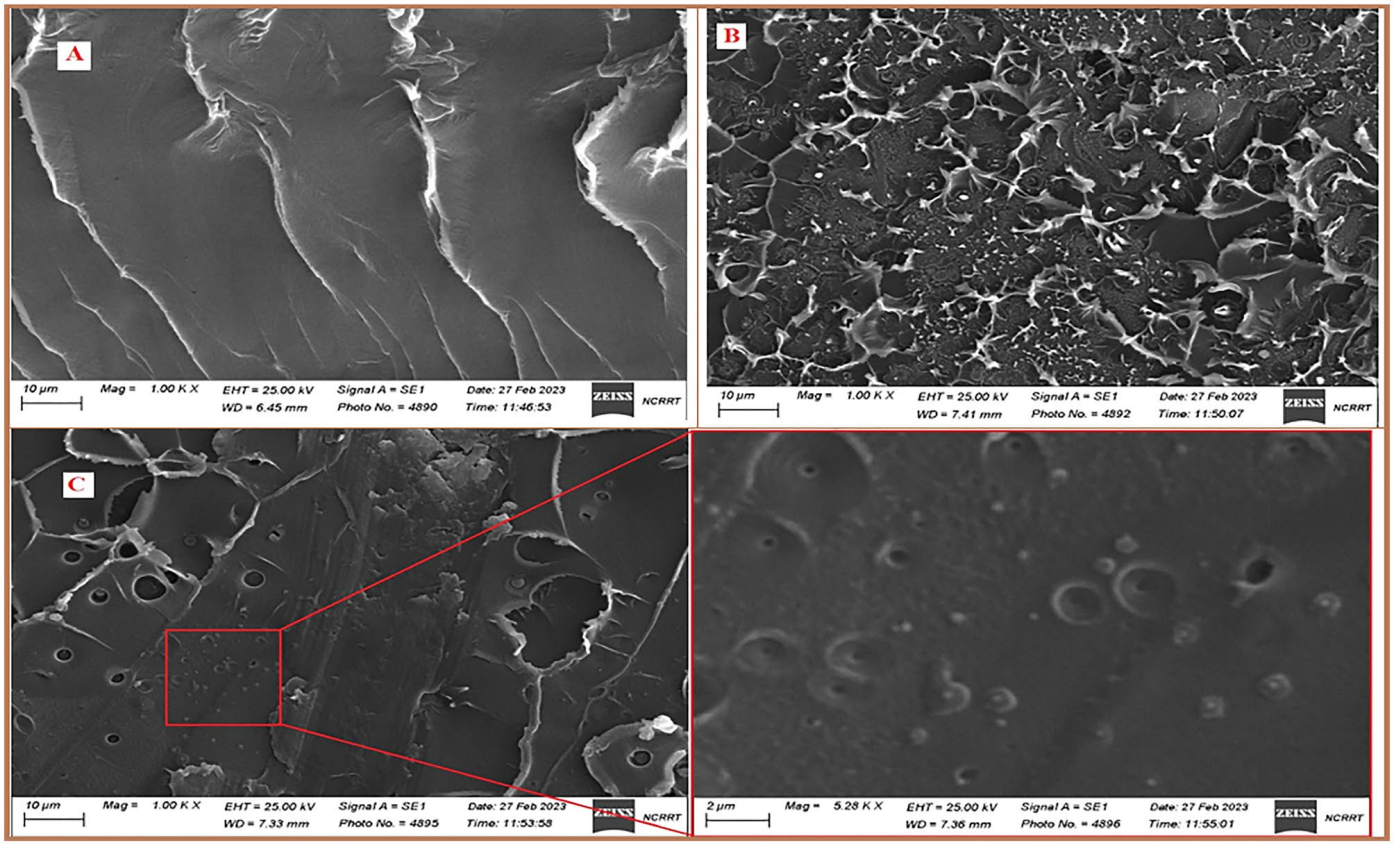
SEM images of (**A**) PS polymer, (**B**) Unirradiated PS/PANI-Cu (3%) nanocomposite film and (**C**) Irradiated PS/ PANI-Cu (3%) nanocomposite film.

The SEM images of PS polymer and PS/PANI-Cu nanocomposite films are shown in Fig. 9. It can be observed that, PANI-Cu nanoparticles are uniformly dispersed within PS polymer. Also, the surface roughness for gamma irradiated PS/PANI-Cu nanocomposite films decreases, indicating radiation crosslinking process.

### Electromagnetic shielding effectiveness

The attenuation of the propagation of an incident electromagnetic wave defines the idea of electromagnetic shielding property. The attenuation of these waves can be happen due to different mechanisms namely the reflection, absorption and multiple reflections. The power ratio between the incident and transmitted electromagnetic waves represents its shielding effectiveness (SE). The total shielding effectiveness (SE) is computed as the sum of the reflected shielding effectiveness (SE_R_), the absorption shielding effectiveness (SE_A_), and the multiple shielding effectiveness (SE_MR_) can be written as: *SE* = *SE*_*R*_ + *SE*_*A*_ + *SE*_*MR*_, in which the third term is very small and can be neglected.

The s-parameter measured using the vector network analyzer, are related to the reflection and transmission coefficient as follows: the transmission (T) equals the squared of the absolute values of S_12_ or S_21_ and the refection (R) equals the squired of the absolute value of S_11_ or S_22_. The total shielding effectiveness can be calculated as the sum of the refection and absorption values and related to the S-parameters as^75^:

$$SE=10\, \text{log}\left(\frac{{P}_{I}}{{P}_{T}}\right)=20\, \text{log}\left(\frac{{E}_{I}}{{E}_{T}}\right)= -10\, \text{log}{\left|{S}_{12}\right|}^{2}=-10\, {\text{log}}_{10}{\left|{S}_{21}\right|}^{2}$$

**Figure 10:**
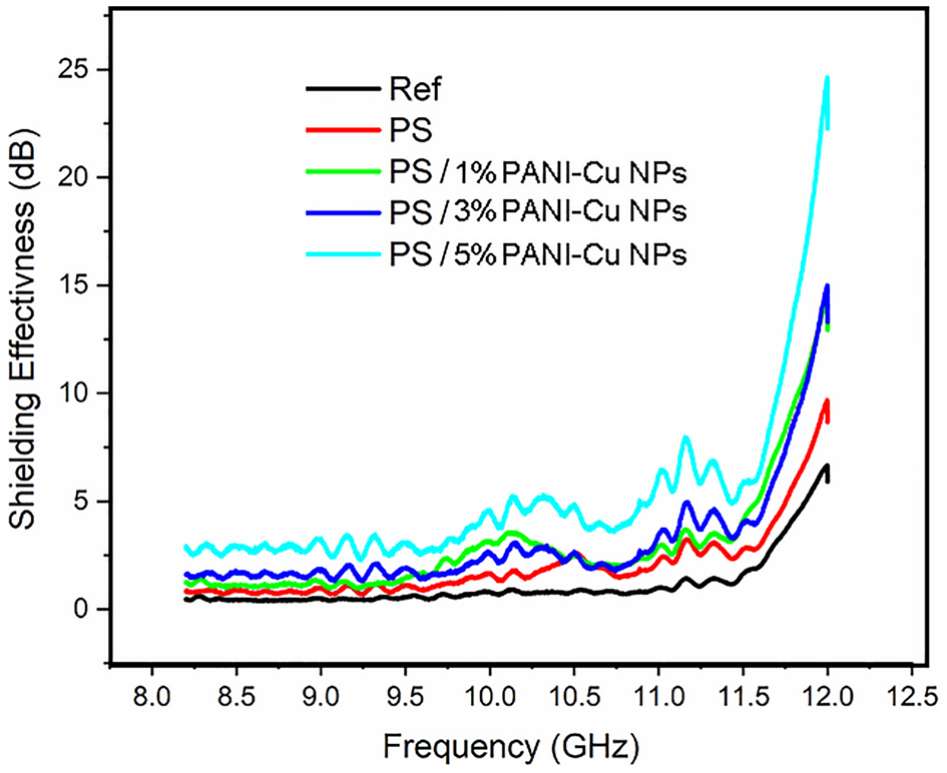
represents the Shielding Effectiveness in (dB) vs. frequency in (GHz) for the unirradiated samples.

The electromagnetic Shielding effectiveness of all prepared samples was measured in the X-band range from 8 to 12 GHz. The response of all samples behaves the same pattern as each one has approximately flat response with its maximum value appears around 11.25 GHz. The second peak at 12 GHz cannot be considered as a peak because it represents the response of the used wave guide as can be seen from the response of the reference line represented by the black line in Fig. 10 for the wave guide without the material.

The shielding effectiveness of the prepared samples before irradiations is presented in Fig. 10. this figure shows an approximately flat response over the frequency range with a small flat peak starts at 10.0 GHz and extends to 10.5 GHz and a peak around 11.25 GHz approximately.

**Figure 11:**
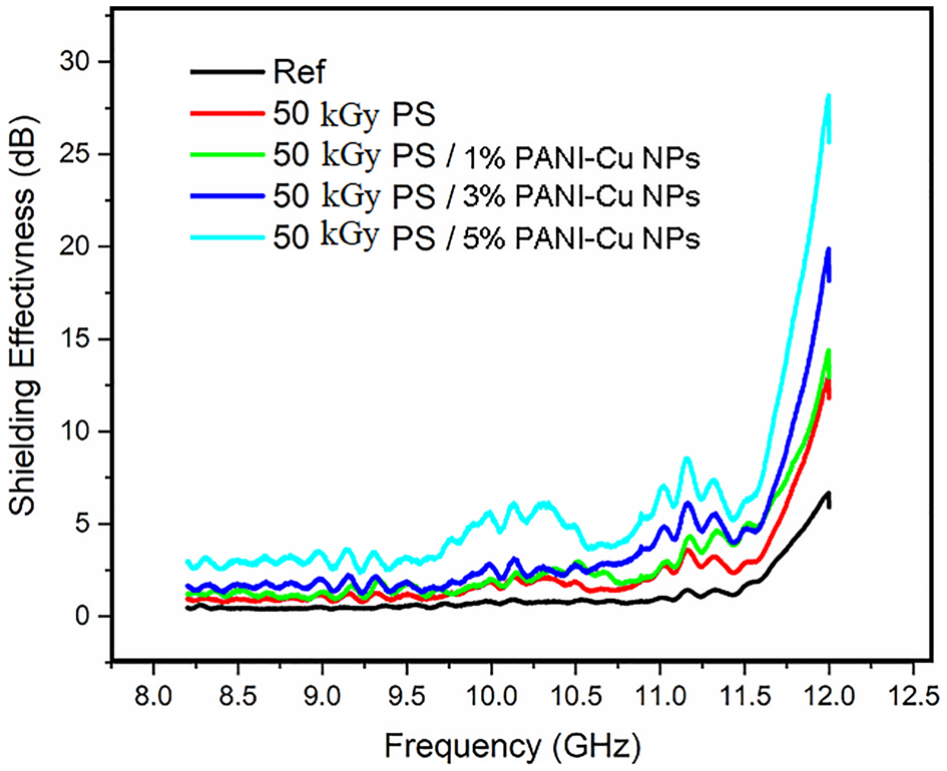
Represents the shielding effectiveness in (dB) versus frequency in (GHz) for 50 kGy gamma-ray irradiated samples.

The shielding effectiveness of the prepared samples after 50 kGy gamma-ray irradiations is presented in Fig. 11. As seen this figure shows an approximately flat response over the frequency range with a small flat peak starts at 10.0 GHz and extends to 10.5 GHz and a peak around 11.25 GHz approximately like that of unirradiated samples with little improvements. This is because gamma irradiation increases the number of free electrons which enhance the electric conductivity significantly that enhancing EMI shielding process^76–79^. The electrical conductivity plays a vital role in the shielding effectiveness mechanisms as it is responsible of the reflection and absorption of the electromagnetic waves. Table 2: summarize the DC conductivity of blank Ps and PANI-Cu NPs with different concentrations. From Table 2, the conductivity increased significantly by increasing the concentrations of PANI-Cu NPs and after irradiation. Addition of PANI-Cu NPs as a nanofiller leads to the creation of numerous conduction paths that means the narrower width of the potential barrier in the bulk region. On the other hand, the irradiation makes the bond between filler and polymer more strong. The change of polymer structure to a carbon-rich network by irradiation makes polymer nanocomposites more conductive^80^.

**Table 2:** DC conductance of the prepared samples before and after 50 KGy gamma-ray irradiations.

Sample	Blank Ps (µS)	1% PANI-Cu NPs (µS)	3% PANI-Cu NPs (µS)	5% PANI-Cu NPs (µS)
Unirradiated	29.373	52.632	78.625	93.173
50 KGy	36.624	69.736	87.564	117.462

**Figure 12:**
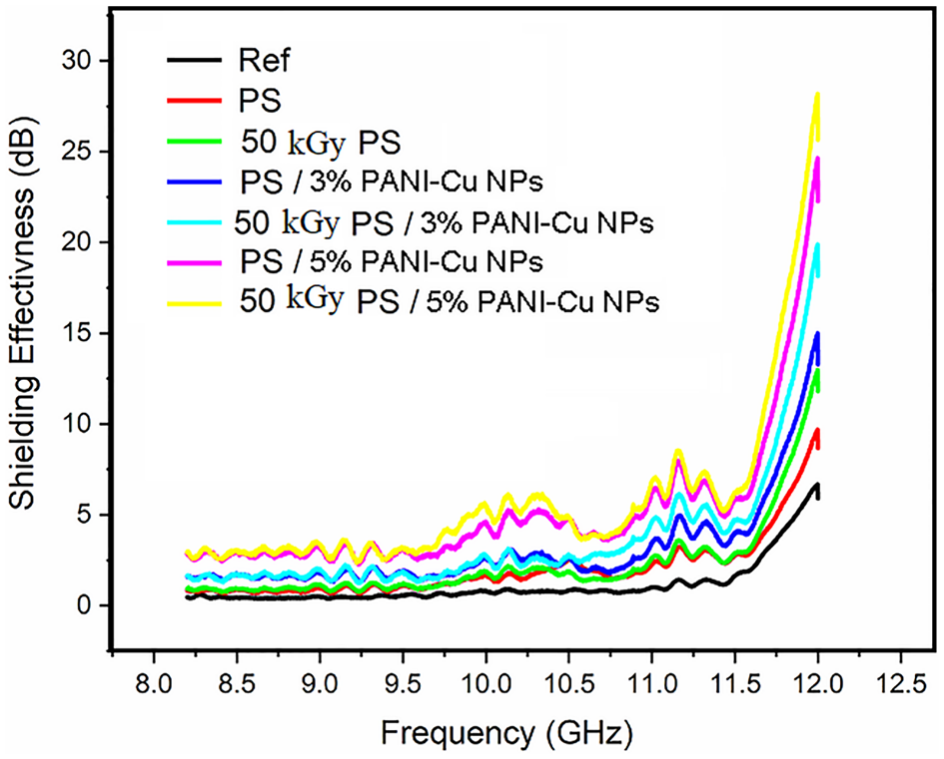
Represents the shielding effectiveness in (dB) versus frequency in (GHz) for unirradiated and 50 kGy gamma-ray irradiated for samples PS, PS/3%, PS/5% to combine the effect of concentration and radiation dose.

The shielding effectiveness for some selected samples (PS, PS/5%, PS/7%) before and after 50 kGy gamma-ray irradiations as a confirmed response was shown in Fig. 12. It is clear from this figure that the SE enhances as the concentration increases and exposure to radiation dose.

## Conclusion

This article presented the synthesis and addition of PANI-Cu NPs in polystyrene polymer with different concentration to prepare PS/PANI-Cu nanocomposites films and applying it as an electromagnetic shielding material to decrease the increasing serious radiation pollution. TEM and XRD investigations proved that the PANI-Cu NPs was successfully formed with particle size equal 30–35 nm. Based on the mechanical results, we conclude that the PANI-Cu NPs positively tensile test results on PS matrix at 3% PANI-Cu NPs and 50 kGy. These characteristics directly affected on the shielding effectiveness properties of the prepared samples. The shielding effectiveness was measured for all samples before and after irradiations with gamma-ray. It is clear that the SE increase significantly with the increase of the concentration of PANI-Cu NPs and exposure to gamma ray.

## Data Availability

All data generated or analyzed during this study are available from the corresponding author on request.
